# Efficacy and Treatment-Related Adverse Events of Romidepsin in PTCL Clinical Studies: A Systematic Review and Meta-Analysis

**DOI:** 10.3389/fmed.2021.732727

**Published:** 2021-11-05

**Authors:** Jun Du, Xinle Han, Suwen Lin, Chen Qiu, Lijun Zhu, Zoufang Huang, Jian Hou

**Affiliations:** ^1^Department of Hematology, Renji Hospital, School of Medicine, Shanghai Jiao Tong University, Shanghai, China; ^2^Biomedical Research Institute, Shenzhen Peking University-The Hong Kong University of Science and Technology Medical Center, Shenzhen, China; ^3^State Key Laboratory of Experimental Hematology, Institute of Hematology and Blood Disease Hospital, Chinese Academy of Medical Sciences and Peking Union Medical College, Tianjin, China; ^4^Binjiang College of Nanjing University of Information Engineering Information Management and System, Wuxi, China; ^5^The First Affliated Hospital of Gannan Medical University, Ganzhou, China

**Keywords:** romidepsin, PTCL, efficiency, adverse events, systematic review, meta-analysis

## Abstract

**Background:** Peripheral T-cell lymphoma (PTCL) is an extensive class of biologically and clinically heterogeneous diseases with dismal outcomes. The histone deacetylase inhibitor (HDACi) romidepsin was approved for relapsed and refractory (R/R-PTCL) in 2011. This meta-analysis was performed to assess the efficacy and safety of romidepsin in PTCL.

**Methods:** We searched for articles on the HDAC inhibitor romidepsin in the treatment of PTCL in Embase, Web of Science, and PubMed. The methodology is further detailed in PROSPERO (CRD42020213651, CRD42020213553). The 2-year overall survival (OS), 2-year progression-free survival (PFS), and their corresponding to 95% confidence intervals (CIs) were measured. Besides, corresponding 95% CIs were pooled for the complete response (CR), partial response (PR), duration of response (DoR), and risk of adverse events (AEs).

**Results:** Eleven studies containing 388 patients were incorporated into the quantitative synthesis, of which R/R-PTCL patients were the dominant portion, accounting for 94.3% (366/388). For all studies, the CR rate was 20% (95% CI, 13–27%, random effects model), and the PR rate was 18% (95% CI, 12–25%, random effects model). The 2-year OS was 48% (95% CI, 38–59%, fixed effects model), and the 2-year PFS was 17% (95% CI, 13–21%, fixed effects model). There were no significant differences between romidepsin monotherapy and romidepsin plus additional drugs. Hematological toxicities, such as lymphopenia and granulocytopenia, remained the most continually happening grade 3 or higher AEs, accounting for 46 and 28%, respectively. None of the studies reported any drug-related mortality.

**Conclusions:** Considering that most of the included patients had R/R-PTCL, the addition of romidepsin significantly enhance the efficacy. And AEs were tolerable as the grade 3/4 AEs in romidepsin monotherapy was 7% (95% CI, 6–8%). It is imperative to further expand the first-line application of romidepsin and carry out personalized therapy based on epigenomics, which will improve the survival of PTCL patients.

**Systematic Review Registration:** https://www.crd.york.ac.uk/prospero/display_record.php?ID=CRD42020213651 and https://www.crd.york.ac.uk/prospero/display_record.php?ID=CRD42020213553.

## Background

Peripheral T-cell lymphoma (PTCL) is a group of malignant tumors with heterogeneous morphological changes in mature T and NK cells and epigenetic alterations that are characterized by dismal outcomes (1). According to the WHO classification of lymphoid neoplasms in 2017, PTCL comprises 29 distinct histological entities. The most common subtype, PTCL not otherwise specified (PTCL-NOS), represents ~25% of cases (2–4). Standard first-line treatment for PTCL includes cyclophosphamide, doxorubicin, vincristine, and prednisone (CHOP) combined with autologous stem cell transplantation (ASCT), but the results are unsatisfactory. Previous studies have shown that PTCL outcomes remain poor, especially in the R/R setting (3, 5), with a median progression-free survival (PFS) of 3.1 months and median overall survival (OS) of 5.5 months (6). Therefore, optimizing and updating the treatment strategy of PTCL is urgent.

Recently, some novel agents have been performed to be promising for the treatment of PTCL, such as histone deacetylase inhibitors (HDACis). The HDAC family is classified into four enzyme classes (I, IIa, IIb, and III), involving 11 HDACs (7). The acetylation stage of histones is extremely important in the development of many malignant lymphomas, and the epigenetic regulation of gene expression is strongly associated with many cellular mechanisms, such as DNA damage, the cell cycle, apoptosis, and immunoregulation (8–10). The classic mechanism of action of HDACis is inhibiting deacetylases and making lysine less electrostatically attractive to DNA, leaving chromatin in an open state and activating the transcription of genes that suppress cell growth or induce a differentiated phenotype (7, 11). The efficacy of HDACis in lymphoma depends on the host immune system and apoptosis, and the mechanisms include interrupting the PI3K/AKT signaling pathway, regulating the expression of BCL-2 family proteins, regulating cell cycle-associated proteins, enhancing the activity of tumor suppressors such as p53 and downregulating the expression of transcriptional repressors such as BCL-6 by acetylation (12–15). Additionally, HDACis can increase the sensitivity of tumor cells to cytotoxic lymphocyte killing by enhancing the phagocytosis of dendritic cells (DCs) and upregulating the expression of costimulatory molecules, NK cell-activating ligands, and MHC class I plus II molecules (12).

Romidepsin (FK228, depsipeptide) is a potent, bicyclic class 1 HDACi with a cycle peptide structure that shows antitumor activity by arresting the cell cycle, increasing apoptosis, and inhibiting angiogenesis (16). In 2011, romidepsin received FDA approval for the treatment of R/R-PTCL, with an ORR of 38% and a median DoR of 8.9 months (17, 18).

To date, some clinical trials have demonstrated the efficiency and safety of romidepsin in R/R-PTCL patients. We performed this systematic review and meta-analysis to investigate the efficacy and safety of romidepsin, providing reliable evidence to optimize the outcome of PTCL patients.

## Methods

### Search Methods and Study Selection

The study search was performed in databases according to the search strategy. All studies were evaluated independently by three investigators (JD, SWL, and XLH), and qualified studies were selected. Studies were searched in PubMed, Embase, and Web of Science with the search terms “Peripheral T-cell Lymphoma, PTCL, Refractory or Relapsed Peripheral T-cell Lymphoma, R/R-PTCL”; “Romidepsin”; “HDAC inhibitors”; “therapeutic effect/effectiveness/efficacy”; “adverse events”; and “treatment.” We limited the search to English language studies on retrieval, and the retrieval time was until February 2021.

After removing duplicated studies via EndNote X9 software and screening the titles, abstracts, and full texts of all eligible studies, we used the following standards to select studies for inclusion: (1) clinical studies involving controlled trials and retrospective studies with large samples (>10 cases) (case reports, letters, reviews, and conference abstracts were excluded); (2) sufficient data on efficacy and adverse events (AEs); (3) patients were treated with romidepsin; (4) the cancer type of patients included PTCL; and (5) English publications. This study followed the Preferred Reporting Items for Systematic Reviews and Meta-Analyses (PRISMA) reporting guidelines and was registered in PROSPERO (CRD42020213651, CRD42020213553) (19).

### Data Extraction and Quality Assessment

Data extraction was accomplished independently by a data analyst (SWL). Any discrepancies were examined by an investigator on the team (XLH) and settled by consensus. The following data were extracted: (1) basic research information, including the study type, author name, regions, year of publication, and number of patients; (2) main characteristics, including the trial phase, PTCL subtypes, drugs, treatment period, number of patients, ages of patients, number of all AEs, and median follow-up time; and (3) main outcomes, including complete response (CR), partial response (PR), DoR, OS, PFS, treatment-related mortality (TRM), all-grade treatment-related AEs and grade 3 or higher AEs. If outcomes were not reported in the article but Kaplan-Meier curves contained the percentage and time for OS or PFS, we used graph digitizer software (Engauge Digitizer, version 11.1) to extract the coordinates of points on the curve and rebuilt the survival data of the included studies by a numeric algorithm.

Quality evaluation was evaluated separately by two investigators (JD, SWL), and the Methodological index for non-randomized studies (MINORS) was used (20). Each study was assessed based on 8 items (a clearly stated aim, the inclusion of consecutive patients, prospective collection of data, endpoints appropriate to the aim of the study, unbiased assessment of the study endpoint, follow-up period appropriate to the aim of the study, loss to follow-up <5%, and prospective calculation of the study size). Each item was scored 0 (not reported), 1 (reported but inadequate) or 2 (reported and adequate). Quality was evaluated based on the total score (<10, low; 10–11, moderate; >11, high). The overall certainty of the evidence for each outcome was assessed via the Grading Recommendations Assessment, Development and Evaluation (GRADE), and any disagreements for the GRADE assessment were settled by consensus. We used the Guideline Development Tool (https://gradepro.org/) to formulate the assessment table.

### Statistical Analysis

This statistical analysis was performed by R studio software (version 4.0.3) using the metafor package (version 2.4-0) and the meta package (version 4.15-1). Study effect sizes were modeled as proportions, in which the numerator was the number of patients meeting the corresponding outcome measure criteria, and the denominator was the total number of enrolled patients. A weighted fixed effects model and random effects model were used to determine each outcome measure's pooled rate, described on a forest plot with the 95% CI and CI interval of proportion.

We analyzed the CR, PR, 2-year OS, and 2-year PFS rates of patients treated with romidepsin or romidepsin plus other drugs. The heterogeneity among studies was assessed by the *I*^2^ statistic, and the *p* value indicated variation across pooled estimates likely associated with statistical heterogeneity (*p* < 0.05 was considered statistically significant). *I*^2^ values of ~25%, 26–75%, 76–100% were considered low, moderate and high, respectively. The potential source of heterogeneity was explored by sensitivity analysis, and alternative methods such as contour-enhanced funnel plots and different models were used depending on the magnitude of heterogeneity; a fixed effects model was used for studies with *I*^2^ ≤ 50%, and a random effects model was used for studies with *I*^2^>50%. Besides, we assessed the presence of publication bias by funnel plots, Egger's test, Begg's test, Peters' test, and the trim & fill method.

## Results

### Eligible Studies and Characteristics

**Figure 1:**
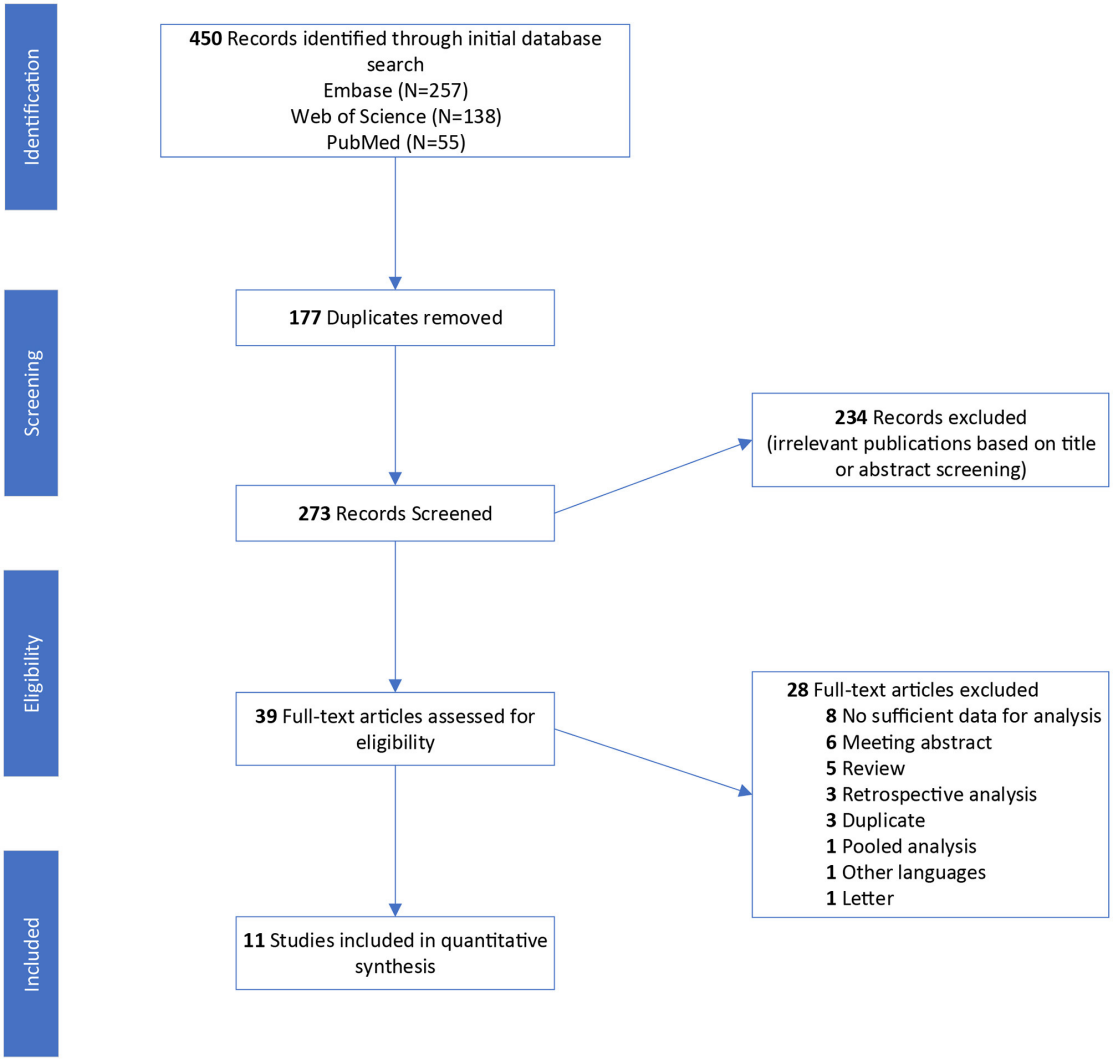
Flow diagram of the study selection process.

In total, 450 studies were identified with the initial search strategy. After removing 177 duplications and excluding 234 irrelevant publications, 39 articles were assessed in full text. According to the study selection criteria, eleven studies involving 388 patients were included in the systematic review and qualitative synthesis (18, 21–30) (Figure 1). Among these studies, one study (9.1%) was a retrospective study (30) and ten studies (90.9%) were clinical trials, including two randomized clinical trials (RCTs) (18, 26), three non-randomized clinical trials (NCTs) (21–23) and five unspecified types of clinical trials (24, 25, 27–29). Five studies (45.5%) (18, 26–28, 30) used romidepsin monotherapy in R/R-PTCL, and six studies (54.5%) (21–25, 29) treated R/R-PTCL with romidepsin combined with additional drugs. The overall summary characteristics of these eleven studies and studies for AEs are shown in Tables 1, 2, respectively.

**Table 1:** Characteristics of the included studies.

**References (sample size)**	**Country**	**Study period**	**Study types (identifier)**	**Drugs**	**PTCL subtypes**	**Median age, years (range)**	**Treatment period**	**Outcome measure**						**Follow-up**
								**CR**	**PR**	**DOR**	**OS**	**PFS**	**TRM**	
Piekarz et al. (18) (*N* = 47)	U.S.	NA	RCT II (NCT00007345)	R	PTCL-NOS (*N* = 27) AITL (*N* = 2) Others (*N* = 18)	59 (27–84)	12 w	8/47 (17%)	9/47 (19%)	8.9 m	NA	NA	NA	NA
Coiffier et al. (27) (*N* = 131)	U.S.	2009-2010	CT II (GPI-06-0002, NCT00426764)	R	PTCL-NOS (*N* = 69) AITL (*N* = 27) ALCL, ALK- (*N* = 21) Others (*N* = 13)	61 (20–83)	24 w	19/130 (10%)	14/130 (11%)	17 m	NA	4 m	1/130 (0.8%)	13.4 m
Pellegrini et al. (25) (*N* = 20)	Italy	2013–2014	CT II (NCT01822886)	R+G	PTCL-NOS (*N* = 10) AITL (*N* = 9) ALCL, ALK- (*N* = 1)	55 (24–77)	24 w	3/20 (15%)	3/20 (15%)	12.4 m	22 m	2.5 m	NA	18 m
Maruyama et al. (28) (*N* = 50)	Japan	NA	CT I/II (NCT01456039)	R	PTCL-NOS (*N* = 20) AITL (*N* = 21) ALCL, ALK- (*N* = 3) Others (*N* = 4)	70 (43–83)	12.9 w	13/50 (26%)	11/50 (22%)	11.1 m	11.1 m	5.6 m	0	NA
Amengual et al. (23) (*N* = 29)	U.S.	2017–2018	NCT I (NCT01947140)	R+P	PTCL-NOS (*N* = 1) ALCL, ALK- (*N* = 3) ATLL (*N* = 6) Others (*N* = 8)	54 (23–73)	16 w	4/18 (22%)	6/18 (33%)	4.29 m	12.4 m	4.4 m	0	NA
O'Connor et al. (26) (*N* = 271)	U.S.	2012–2014	RCT III (NCT01482962)	R	PTCL-NOS (*N* = 12) AITL (*N* = 6) ALCL, ALK- (*N* = 3) Others (*N* = 2)	63 (44–80)	16.6 w	4/23 (17%)	7/23 (30%)	15.8 m	12.2 m	3.47 m	0	19.5 m
O'Connor et al. (22) (*N* = 31)	U.S.	2013–2017	NCT I/II (NCT01998035)	5-A+R	PTCL-NOS (*N* = 2) AITL (*N* = 3) ALCL (*N* = 1) Others (*N* = 5)	57 (23–79)	NA	6/11 (55%)	2/11 (18%)	NA	NA	NA	0	15.3 m
Reiman et al. (24) (*N* = 20)	Canada	2013–2016	CTI (NCT01846390)	R+G+D+Ch	PTCL-NOS (*N* = 5) ALCL, ALK+ (*N* = 1) ATLL (*N* = 2) Others (*N* = 2)	65 (24–74)	9.2 w	0	6/10 (60%)	2.8 m	15.8 m	5.45 m	0	9.7 m
Shimony et al. (30) (*N* = 42)	Israel	2013–2018	Retrospective study	R	PTCL-NOS (NA) AITL (*N* = 6) ALCL, ALK+ (*N* = 2) Others (*N* = 6)	66 (25–82)	NA	5/42 (13%)	8/42 (20%)	13.4 m	7.1 m	2.2 m	NA	6.2 m
Falchi et al. (21) (*N* = 25)	U.S.	2017–2019	NCT II (NCT01998035)	5-A+R	PTCL-NOS (*N* = 4) PTCL-TFH (*N* = 3) AITL (*N* = 14) ALCL (*N* = 1) Others (*N* = 3)	63 (42–88)	30 w	10/25 (40%)	4/25 (16%)	20.3 m	NA	8 m	0	13.5 m
Vu et al. (29) (*N* = 24)	U.S.	NA	CTI (NCT01902225)	R+L	PTCL-NOS (*N* = 5) AITL (*N* = 4) ALCL, ALK- (*N* = 2) Others (*N* = 1)	63 (52–83)	32 w	3/12 (25%)	0	4.2 m	17.5 m	2.1 m	NA	17.8 m

*CR, complete response; PR, partial response; DOR, duration of response; OS, overall survival; PFS, progression-free survival; RCT, randomized clinical trial; NCT, non-randomized clinical trial; CT, clinical trial; NA, not available; R, romidepsin, Ci, cisplatin; Ch, chop; G, gemcitabine; P, pralatrexate; 5-A, 5-azacytidine; L, liposomal doxorubicin; w, weeks; m, months*.

**Table 2A:** Characteristics of the included studies for treatment-related adverse events.

**References (sample size)**	**Country**	**Study period**	**Study types (identifier)**	**Drugs**	**PTCL subtypes**	**Median age, years (range)**	**Treatment period**	**Follow-up**
Piekarz et al. (18) (*N* = 47)	U.S.	NA	RCT II (NCT00007345)	R	PTCL-NOS (*N* = 27) AITL (*N* = 2) Others (*N* = 18)	59 (27–84)	12 w	NA
Coiffier et al. (27) (*N* = 131)	U.S.	2009–2010	CT II (GPI-06-0002, NCT00426764)	R	PTCL-NOS (*N* = 69) AITL (*N* = 27) ALCL, ALK- (*N* = 21) Others (*N* = 13)	61 (20–83)	24 w	13.4 m
Pellegrini et al. (25) (*N* = 20)	Italy	2013–2014	CT II (NCT01822886)	R+G	PTCL-NOS (*N* = 10) AITL (*N* = 9) ALCL, ALK- (*N* = 1)	55 (24–77)	24 w	18 m
Maruyama et al. (28) (*N* = 50)	Japan	NA	CT I/II (NCT01456039)	R	PTCL-NOS (*N* = 20) AITL (*N* = 21) ALCL, ALK- (*N* = 3) Others (*N* =4)	70 (43–83)	12.9 w	NA
Amengual et al. (23) (*N* = 29)	U.S.	2017–2018	NCT I (NCT01947140)	R+P	PTCL-NOS (*N* = 1) ALCL, ALK- (*N* = 3) ATLL (*N* = 6) Others (*N* = 8)	54 (23–73)	16 w	NA
Shimony et al. (30) (*N* = 42)	Israel	2013–2018	Retrospective study	R	PTCL-NOS (NA) AITL (*N* = 6) ALCL, ALK+ (*N* = 2) Others (*N* = 6)	66 (25–82)	NA	6.2 m
Falchi et al. (21) (*N* = 25)	U.S.	2017–2019	NCT II (NCT01998035)	5-A+R	PTCL-NOS (*N* = 4) PTCL-TFH (*N* = 3) AITL (*N* = 14) ALCL (*N* = 1) Others (*N* = 6)	63 (42–88)	30 w	13.5 m

*R, romidepsin, Ci, cisplatin; Ch, Chop; G, gemcitabine; P, pralatrexate; 5-A, 5-azacytidine; L, liposomal doxorubicin; w, weeks; m, months*.

**Table 2B:** Treatment-related adverse events.

	**Romidepsin monotherapy (*****N*** **=** **250)**		
**Adverse events**	**All grade**	**Grade <3**	**Grade≥3**
**GRADE NO. (%)**			
**Hematologic**			
Thrombocytopenia	152 (60.8)	94 (37.6)	58 (23.2)
Neutropenia	113 (45.2)	58 (23.2)	55 (22.0)
Anemia	75 (33.2)	58 (23.3)	17 (18.8)
Leukopenia	92 (36.8)	45 (18.0)	47 (18.8)
Lymphopenia	52 (20.8)	7 (2.8)	45 (18.0)
Granulocytopenia	22 (8.8)	9 (3.6)	13 (5.2)
Infection w/o neutropenia	3 (1.2)	2 (0.8)	1 (0.4)
**Laboratory**			
Hypoalbuminemia	10 (4.0)	9 (3.6)	1 (0.4)
Hypokalemia	14 (5.6)	12 (4.8)	2 (0.8)
Decreased hemoglobin	13 (5.2)	7 (2.8)	6 (2.4)
Hypomagnesemia	5 (2.0)	5 (2.0)	0 (0)
Hypocalcemia	18 (7.2)	17 (6.8)	1 (0.4)
Hyperuricemia	10 (4.0)	7 (2.8)	3 (1.2)
Elevated AST	9 (3.6)	7 (2.8)	2 (0.8)
Hyponatremia	5 (2.0)	5 (2.0)	0 (0.0)
Hyperbilirubinemia	7 (2.8)	6 (2.4)	1 (0.4)
Elevated ALT	7 (2.8)	7 (2.8)	0 (0.0)
Hyperglycemia	7 (2.8)	7 (2.8)	0 (0.0)
Platelet count decrease	6 (2.4)	5 (2.0)	1 (0.4)
**Digestive**			
Nausea	139 (55.6)	136 (54.4)	3 (1.2)
Vomiting	84 (33.6)	78 (31.2)	6 (2.4)
Diarrhea	24 (9.6)	24 (9.6)	0 (0.0)
Anorexia	46 (18.4)	44 (17.6)	2 (0.8)
Constipation	42 (16.8)	42 (16.8)	0 (0.0)
Decreased appetite	38 (15.2)	32 (12.8)	6 (2.4)
Abdominal pain	8 (3.2)	8 (3.2)	0 (0.0)
Stomatitis	12 (4.8)	11 (4.4)	1 (0.4)
Decreased weight	10 (4.0)	10 (4.0)	0 (0.0)
Dysgeusia	61 (24.4)	61 (24.4)	0 (0.0)
**Cardiovascular**			
ECG T-wave changes	30 (12.0)	30 (12.0)	0 (0.0)
Tachycardia	6 (2.4)	6 (2.4)	0
**Respiratory**			
Dyspnea	16 (6.4)	12 (4.8)	4 (1.6)
Cough	5 (2.0)	5 (2.0)	0 (0.0)
Infections			
Infections soc^*^	32 (12.8)	24 (9.6)	8 (3.2)
**Others**			
Fatigue	120 (48)	105 (42)	15 (6.0)
Fever	74 (30.8)	66 (27.6)	8 (3.2)
Headache	22 (8.8)	22 (8.8)	0 (0.0)
Malaise	13 (5.2)	13 (5.2)	0
Peripheral edema	8 (3.2)	8 (3.2)	0 (0.0)
Chills	6 (2.4)	6 (2.4)	0 (0.0)
Pruritus	5 (2.0)	5 (2.0)	0 (0.0)
Dizziness	2 (0.8)	2 (0.8)	0 (0.0)

* *soc, system organ class*.

### Overall Proportions of CR and PR

**Figure 2:**
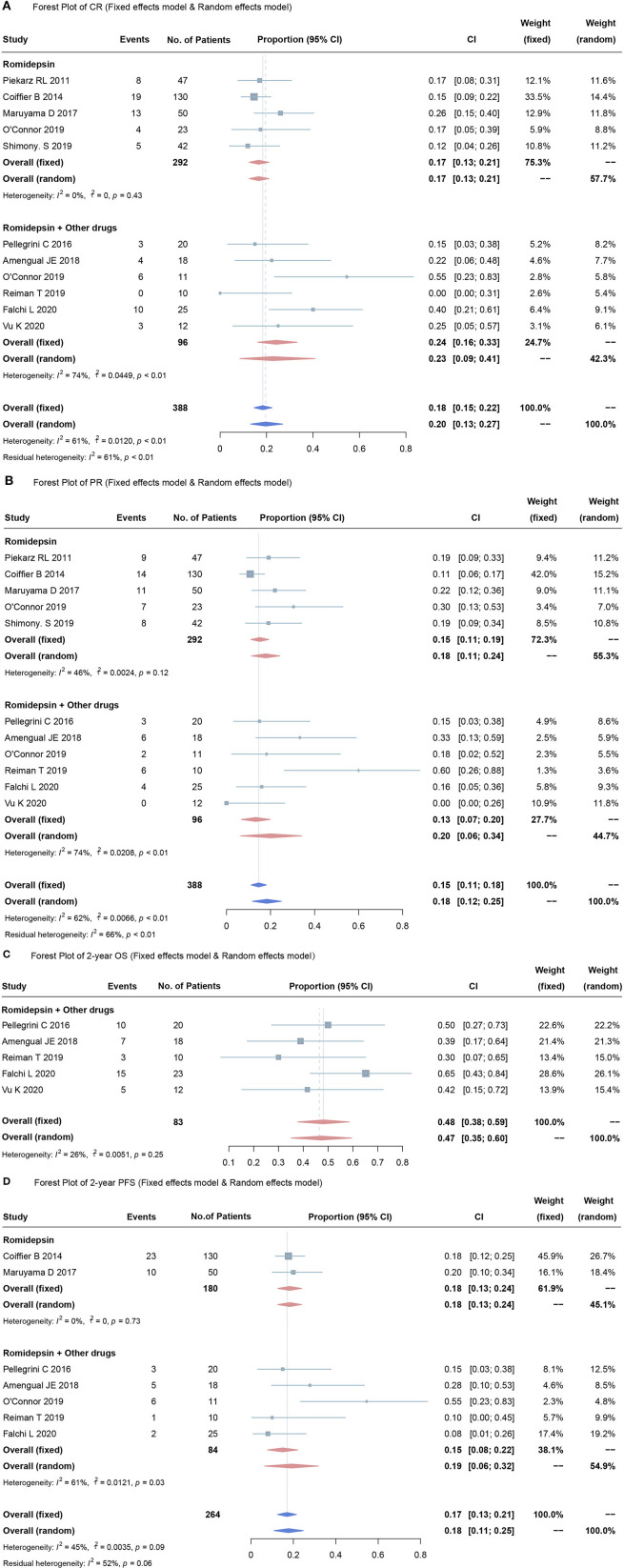
Forest plots for over proportions of CR **(A)**, PR **(B)**, OS **(C)**, PFS **(D)**. Heterogeneity was assessed by using the *I*^2^ statistic (0–25%, low likelihood; 26–75%, moderate likelihood; 76–100%, high likelihood), and the *p* value was used to test the heterogeneity of all studies (*p* < 0.05 indicates likely variation across pooled estimates related to statistical heterogeneity).

Data from all eleven studies were gathered, and the response rates (CR and PR) generated by romidepsin were summarized and evaluated. The CR of all 388 PTCL patients was 20% (95% CI, 13–27%; random effects model, with observed heterogeneity, *I*^2^ = 61%; *p* < 0.01). Of these, five studies included 292 patients who received romidepsin monotherapy (18, 26–28, 30). In the pooled CR analysis, the overall mean proportion was 17% (95% CI, 13–21%; fixed effects model, no significant study heterogeneity, *I*^2^ = 0%; *p* = 0.43). Six studies involved 96 patients treated with romidepsin plus other drugs (21–25, 29). The pooled estimated CR was 23% (95% CI, 9–41%; random effects model, with observed heterogeneity, *I*^2^ = 74%; *p* < 0.01). There was no significant discrepancy in CR when comparing romidepsin monotherapy and romidepsin plus other drugs (*p* = 0.473) (Figure 2A).

Of the eleven studies, the pooled estimated PR was 18% (95% CI, 12–25%; random effects model, with observed heterogeneity, *I*^2^ = 62%; *p* < 0.01). The pooled results showed that when compared to romidepsin monotherapy (PR 15%, 95% CI, 11–19%; fixed effects model, no significant study heterogeneity, *I*^2^ = 46%; *p* = 0.12) (18, 26–28, 30), treatment with romidepsin plus extra medication was associated with no significant difference in terms of the PR rate (PR 20%, 95% CI, 6–34%; random effects model, with observed heterogeneity, *I*^2^ = 74%; *p* < 0.01) (21–25, 29) (Figure 2B).

### Overall Proportions of 2-Year OS and 2-Year PFS

Among the included studies, 2-year OS was reported in five studies, involving 83 patients (21, 23–25, 29). The overall mean proportion was 48% (95% CI, 38–59%; fixed effects model, no significant study heterogeneity, *I*^2^ = 26%; *p* = 0.25) (Figure 2C). The 2-year PFS was reported in seven studies, with a pooled estimated 2-year PFS of 17% (95% CI, 13–21%; fixed effects model, no significant study heterogeneity, *I*^2^ = 45%; *p* = 0.09). Of these, two studies (27, 28) used romidepsin monotherapy and five studies used romidepsin plus other drugs (21–25)]; the 2-year PFS rates were 18% (95% CI, 13–24%; fixed effects model, no significant studies heterogeneity, *I*^2^ = 0%; *p* <0.73) and 19% (95% CI, 6–32%; random effects model, with observed heterogeneity, *I*^2^ = 61%; *p* = 0.03), respectively.

There was no significant discrepancy in the overall proportion of PR between combination therapy and monotherapy (*p* = 0.475) (Figure 2D).

### Publication Bias Test for CR, PR, 2-Year OS, and 2-Year PFS

**Figure 3:**
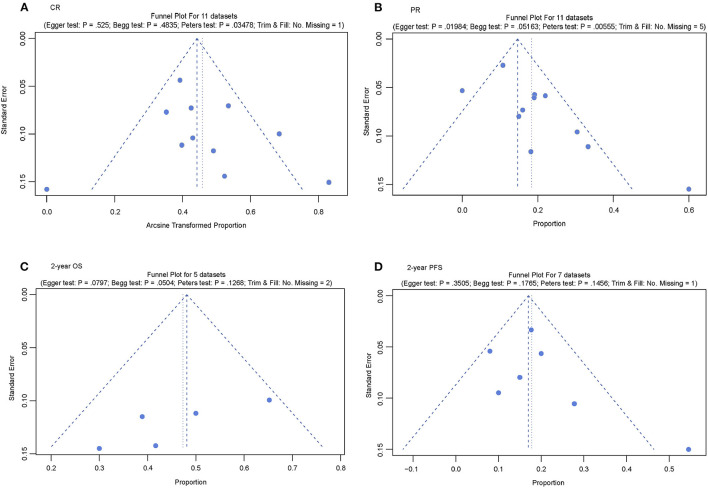
Publication bias test of the included studies. **(A)** Funnel plot for complete response (CR); **(B)** Funnel plot for partial response (PR); **(C)** Funnel plot for 2-year overall survival (OS); and **(D)** Funnel plot for 2-year progression-free survival (PFS).

A funnel plot was used to assess potential publication bias; asymmetry in the funnel plot indicates publication bias. Publication bias was found for the datasets of CR and PR. However, the studies that reported the 2-year OS and 2-year PFS did not show any evidence of publication bias (Figure 3).

### Sensitivity Test and Heterogeneity Analysis of CR, PR, 2-Year OS, and 2-Year PFS

A sensitivity test was used to explore the potential sources of heterogeneity and bias. In the forest plot of CR, the overall mean proportion was 20% (95% CI, 13–27%), four studies [Falchi et al. (21), O'Connor et al. (22), Reiman et al. (24), and Shimony et al. (30) (romidepsin plus other drugs)], five studies [O'Connor et al. (22) (romidepsin), Amengual et al. (23), O'Connor et al. (26) (romidepsin plus other drugs), Coiffier et al. (31), and Vu et al. (29)], one study [Falchi et al. (21)] and two studies [Falchi et al. (21) and O'Connor et al. (22) (romidepsin plus other drugs)] may be the source of potential heterogeneity in the CR, PR 2-year OS, and 2-year PFS datasets, respectively (Supplementary Figure S1).

Additionally, a contour-enhanced funnel plot was used to explore potential bias. Except for the 2-year OS dataset, publication bias existed in the other three datasets. Two datasets (PR and 2-year OS) had other biases in addition to publication bias (Supplementary Figure S2).

### Overall Proportion of Treatment-Related Adverse Events

**Figure 4:**
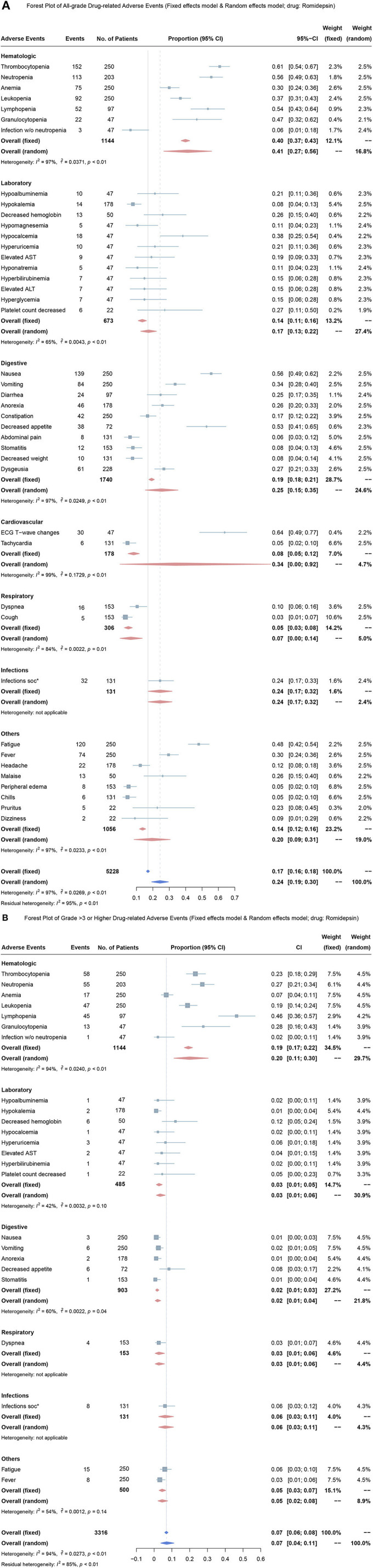
Forest plots for over proportion of adverse events in treatment with romidepsin. **(A)** Proportion of all-grade adverse events in treatment with romidepsin; **(B)** Proportion of grade 3 or higher adverse events in treatment with romidepsin. ALT, alanine aminotransferase; AST, aspartate aminotransferase.

These eleven studies reported 77 different types of treatment-related AEs. Overall, 193 (82.3%) of 234 patients developed grade 1 or 2 AEs, and 116 (49.6%) of 234 patients developed grade 3 or higher AEs. In this meta-analysis, we included the most common AEs in seen clinical practice. Using the random effects model, the overall mean proportion of all-grade AEs in romidepsin monotherapy was 24% (95% CI, 19–30%) (Figure 4A). The overall mean proportion of grade 3 or higher AEs in romidepsin monotherapy was 7% (95% CI, 6–8%) (Figure 4B).

In romidepsin monotherapy, the four most common AEs were ECG-T wave change (64%, 95% CI, 49–77%), thrombocytopenia (61%, 95% CI, 54–67%), neutropenia (56%, 95% CI, 49–63%) and nausea (56%, 95% CI, 49–62%), and the three most common grade 3 or higher AEs were lymphopenia (46%, 95% CI, 36–57%), granulocytopenia (28%, 95% CI, 16–43%), and neutropenia (27%, 95% CI, 21–34%).

The overall mean proportion of all-grade treatment-related adverse events in Romidepsin plus other drugs was 20% (95% CI, 0.04–0.11) (Supplementary Figure S3A), and the overall mean proportion of grade 3 or higher treatment-related adverse events in the treatment of Romidepsin plus other drugs, that was 10% (95% CI, 0.07–0.14) (Supplementary Figure S3B).

In the treatment with Romidepsin plus other drugs, three of the most common all-grade treatment-related adverse events were platelet count decreased (72%, 95% CI, 51–88%), neutrophil count decreased (68%, 95% CI, 0.46–0.85) and nausea (67%, 95% CI, 53–79%) and three of the most common grade 3 or higher treatment-related adverse events were platelet count decreased (48%, 95% CI, 28–69%), neutrophil count decreased (40%, 95% CI, 21–61%) and lymphocyte count decreased (32%, 95% CI, 15–54%).

### Quality Assessment of Outcome Evidence and of the Included Studies

**Figure 5:**
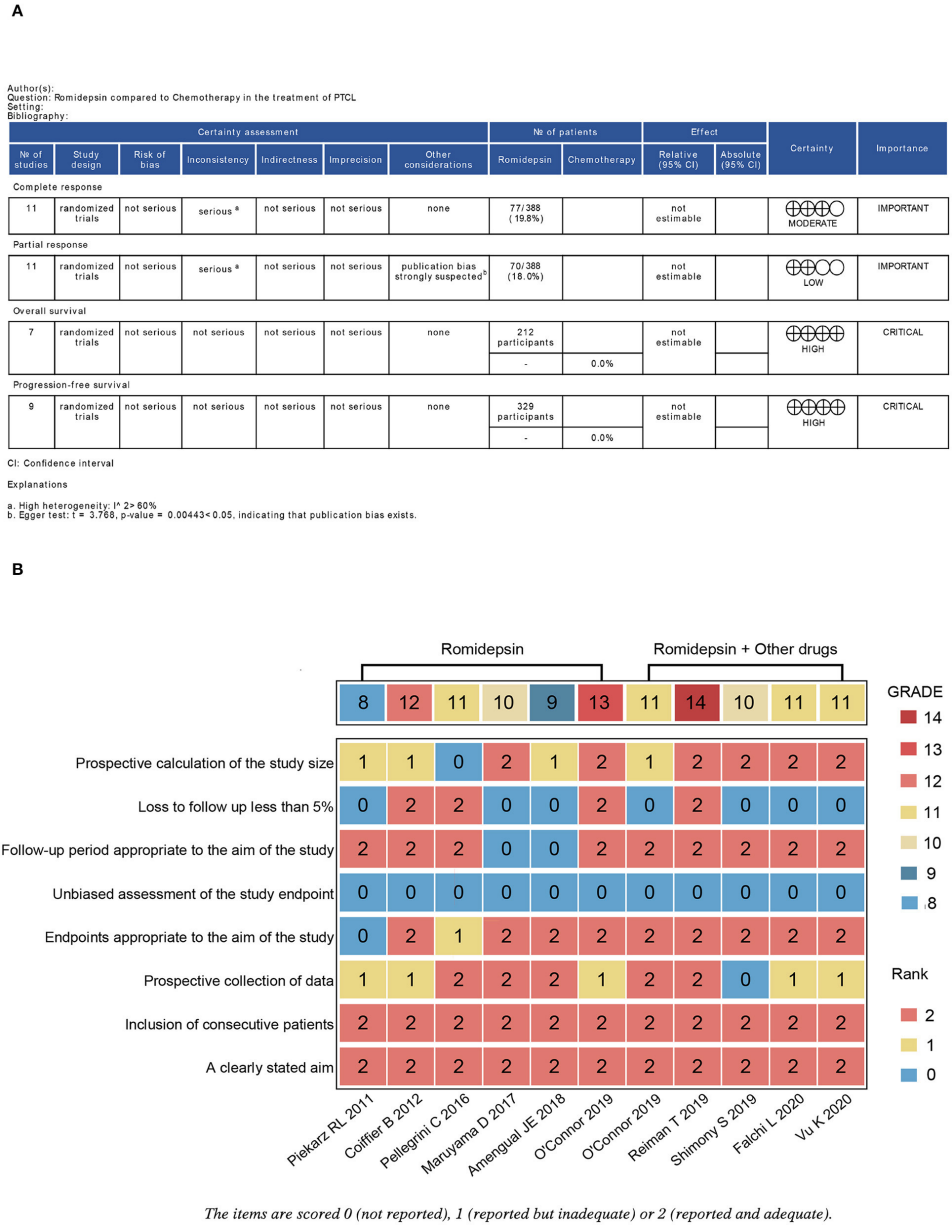
Evaluation of the grade of outcomes and the quality of the included studies. **(A)** Grade analysis table of outcomes; **(B)** Heatmap plot of the quality evaluation for the included studies. The overall certainty of the evidence for outcomes was assessed by the Grading Recommendations Assessment, Development and Evaluation (GRADE) approach.

A summary of each dimension's quality of evidence and assessment according to the outcome is depicted in Figure 5A. The estimated certainties of evidence for PR and CR were assessed as low and moderate, respectively; for OS and PFS, they were evaluated as high. We evaluated the quality of all included studies by MINORS. Three of the included studies were of high quality, including the studies of Reiman et al. (24) (romidepsin), O'Connor et al. (26), and Coiffier et al. (27) (Figure 5B).

## Discussion

PTCL is a wide group of biologically and clinically heterogeneous diseases characterized by dismal outcomes, especially in the R/R setting, with a 5-year OS of ~32%. The therapeutic option in the first-line setting is most often CHOP or CHOP-like regimens. In several reports, a proportion of patients varying from 60 to 85% were treated with this strategy (3, 32, 33). The CHOP combination is effective in B cell lymphomas such as diffuse large B-cell lymphoma (DLBCL); however, its counterpart PTCL is associated with a poor prognosis; chemotherapy alone has resulted in median overall survival (OS) of 6.5 months due to rapid relapse (15, 34).

To improve the inferior outcome of PTCL, therapeutic strategies based on precision medicine are necessary. The most significant progress in recent years has been made by introducing numerous novel agents, including HDAC inhibitors, monoclonal antibodies, immunoconjugates, antifolates, immunomodulatory agents, nucleoside analogs, proteasome inhibitors, kinase inhibitors, and other targeted agents (35). In this work, we focused on an HDAC inhibitor and evaluated its application in PTCL.

This meta-analysis involved 388 PTCL patients and consisted of ten clinical trials and one retrospective study. Among these, R/R-PTCL patients accounted for 94.3% (366/388) and had a universally poor outcome, usually showing resistance to chemotherapy. The CR of all included patients was 20%, and the PR was 18%, demonstrating that romidepsin has favorable efficacy in R/R-PTCL patients. As a kind of epigenetic-based therapy, romidepsin showed encouraging and unique activity in PTCL. As a single-agent treatment for R/R-PTCL, romidepsin had an ORR of 25% and a median DoR of 28 months (27, 31). Additionally, Vu et al. observed that two-thirds of all CR cases occurred in AITL patients, indicating that romidepsin was more effective in AITL patients, probably because AITL carries high-frequency mutations (TET2, RHOA, and DNMT3A) in epigenetic regulators (29). Extensive mutations in epigenetic regulators may be positively related with the response of PTCL to epigenetic therapy (21).

In terms of treatment-related AEs, none of the included studies reported treatment-related mortality. Hematological toxicity occurred more frequently, which may be associated with myelosuppressive therapy. Besides, most non-hematologic AEs were grade 1 or 2, which is consistent with the safety of romidepsin monotherapy in other clinical studies. Although AEs occurred in treatment with romidepsin, they were well-tolerated and managed.

According to a previous study, CHOP and CHOP-like regimens, which are the frontline and backbone therapy of PTCL, produced general toxicities far outweighing the benefits, and long-term reliance on those regimens can lead to a decrease in the long-term OS (36). Therefore, high-dose chemotherapy followed by autologous stem cell transplantation (ASCT) has been accepted as a salvage treatment for eligible patients (37). Romidepsin is considered a supplement to CHOP therapy and is well-tolerated and effective when adopted in R/R-PTCL patients, showing its promising application in PTCL. However, the specific PTCL subtype of patients that would benefit the most remains unidentified in this study, and further research is needed to analyze the efficacy of the Ro-CHOP regimen in different PTCL subtypes. Similar to DNA methyltransferase (DNMT) inhibitors (5-azacytidine), this regimen showed high effectiveness and was well-tolerated in R/R-PTCL, especially in PTCL-tTFH, indicating that romidepsin may also exert efficacy on R/R-PTCL. Likewise, specific groups that would benefit from treatment with romidepsin, an epigenetic drug, should be screened to achieve precision treatment, which should be a direction for future research (21). In parallel, the belinostat plus CHOP (Bel-CHOP) regimen as the first-line treatment for untreated PTCL patients also represented validity, with no additional toxicity (38). This promising finding regarding another epigenetic-based regimen inspired us to explore the epigenomics-based application of romidepsin as first-line treatment with the conventional CHOP regimen for PTCL, as this would be a promising strategy.

Our study objectively evaluates the efficacy and safety of romidepsin and provides a reasonable option for R/R-PTCL. Currently, nine epigenetic drugs have been approved, and most advances in epigenetic therapy for hematologic tumors are still in progress (7). Due to the heterogeneity and rarity of PTCL, especially in the R/R setting, it is challenging to develop a strategy that can comprehensively overcome the inferior outcomes. T cells are widely heterogeneous, and exploration remains promising. Understanding cytokine and T cell functions can greatly promote our understanding and facilitate the precise treatment of immune-related diseases (39). Therefore, efforts are needed to catch up with the drug development progress of B-cell lymphoma and deepen the multidimensional knowledge of pathological T-cell histology. Furthermore, mutations in different epigenetic genes and regulators in different PTCL subtypes need to be identified, and the potential mechanisms also need to be recognized to promote clinical outcomes (8).

In conclusion, romidepsin demonstrated promising efficacy in PTCL patients, especially in the R/R setting. Nonetheless, the precise identification of those who would acquire the most benefits remain to be completed. In the future, romidepsin combined with the CHOP regimen might be a promising first-line treatment strategy for patients with specific PTCL subtypes, which deserves further exploration.

However, there are some limitations in our study. First, the longest median follow-up time was 19.5 months, which may be insufficient to consider all later AEs. Second, owing to the few included studies on 2-year OS and 2-year PFS, publication bias exists. Finally, the reliability of this study remains inconclusive due to the lack of comparability of the included trials. Future studies should focus on these aspects for further research.

## Data Availability Statement

The original contributions presented in the study are included in the article/Supplementary Material, further inquiries can be directed to the corresponding author/s.

## Author Contributions

JH and ZH contributed to the conception and guided the promotion of the whole work. JD and XH designed this study. SL and XH participated in the data collection and analysis. JD, XH, and SL wrote the manuscript and approved the final submission of the study. All authors were involved in reviewing and revising the manuscript and read and approved the final manuscript.

## Funding

This study was funded by the Youth Start-Up Fund for The First Affiliated Hospital of Gannan Medical University (QD081).

## Conflict of Interest

The authors declare that the research was conducted in the absence of any commercial or financial relationships that could be construed as a potential conflict of interest.

## Publisher's Note

All claims expressed in this article are solely those of the authors and do not necessarily represent those of their affiliated organizations, or those of the publisher, the editors and the reviewers. Any product that may be evaluated in this article, or claim that may be made by its manufacturer, is not guaranteed or endorsed by the publisher.

## Supplementary Material

Supplementary Figure S1 Forest plots for sensitivity analysis of CR, PR, 2-year OS and 2-year PFS. **(A)** Forest plot of CR (random effects model); **(B)** Forest plot of PR (random effects model); **(C)** Forest plot of 2-year OS (fixed effects model); **(D)** Forest plot of 2-year PFS (random effects model).

Supplementary Figure S2 Contour-enhanced funnel plots for sensitivity analysis of CR, PR, 2-year OS and 2-year PFS. **(A)** Contour-enhanced funnel plot for complete response (CR); **(B)** Contour-enhanced funnel plot for partial response (PR); **(C)** Contour-enhanced funnel plot for 2-year overall survival (OS); **(D)** Contour-enhanced funnel plot for 2-year progression-free survival (PFS).

Supplementary Figure S3 Forest Plots for Over Proportion of Adverse Events in treatment with romidepsin combined with other drugs. **(A)** Proportion of all-grade adverse events in treatment with romidepsin combined with other drugs; **(B)** Proportion of grade 3 or higher adverse events in treatment with romidepsin combined with other drugs.

## Abbreviations

PTCL: peripheral T-cell lymphoma
R/R PTCL: relapsed/refractory T-cell lymphoma
NHL: non-Hodgkin lymphoma
PTCL-NOS, peripheral T-cell lymphoma: not otherwise specified
AITL: angioimmunoblastic T-cell lymphoma
ALTL, ALK+, anaplastic large-cell lymphoma: ALK-positive
ALTL, ALK-, anaplastic large-cell lymphoma: ALK-negative
ENKTCL: extranodal natural killer or T cell lymphoma
HDACi: histone deacetylase inhibitor
CDKIs: cyclin-dependent kinase inhibitors
ORR: overall response rate
DoR: duration of response
CR: complete response
PR: partial response
OS: overall survival
PFS: progression-free survival
AEs: adverse events
CI: confidence interval.
