# Supplementary figures and images for: Efficacy and Treatment-Related Adverse Events of Romidepsin in PTCL Clinical Studies: A Systematic Review and Meta-Analysis
Source: Front Med (Lausanne). 2021 Nov 5;8:732727. doi: 10.3389/fmed.2021.732727 (PMC8602095; doi:10.3389/fmed.2021.732727)

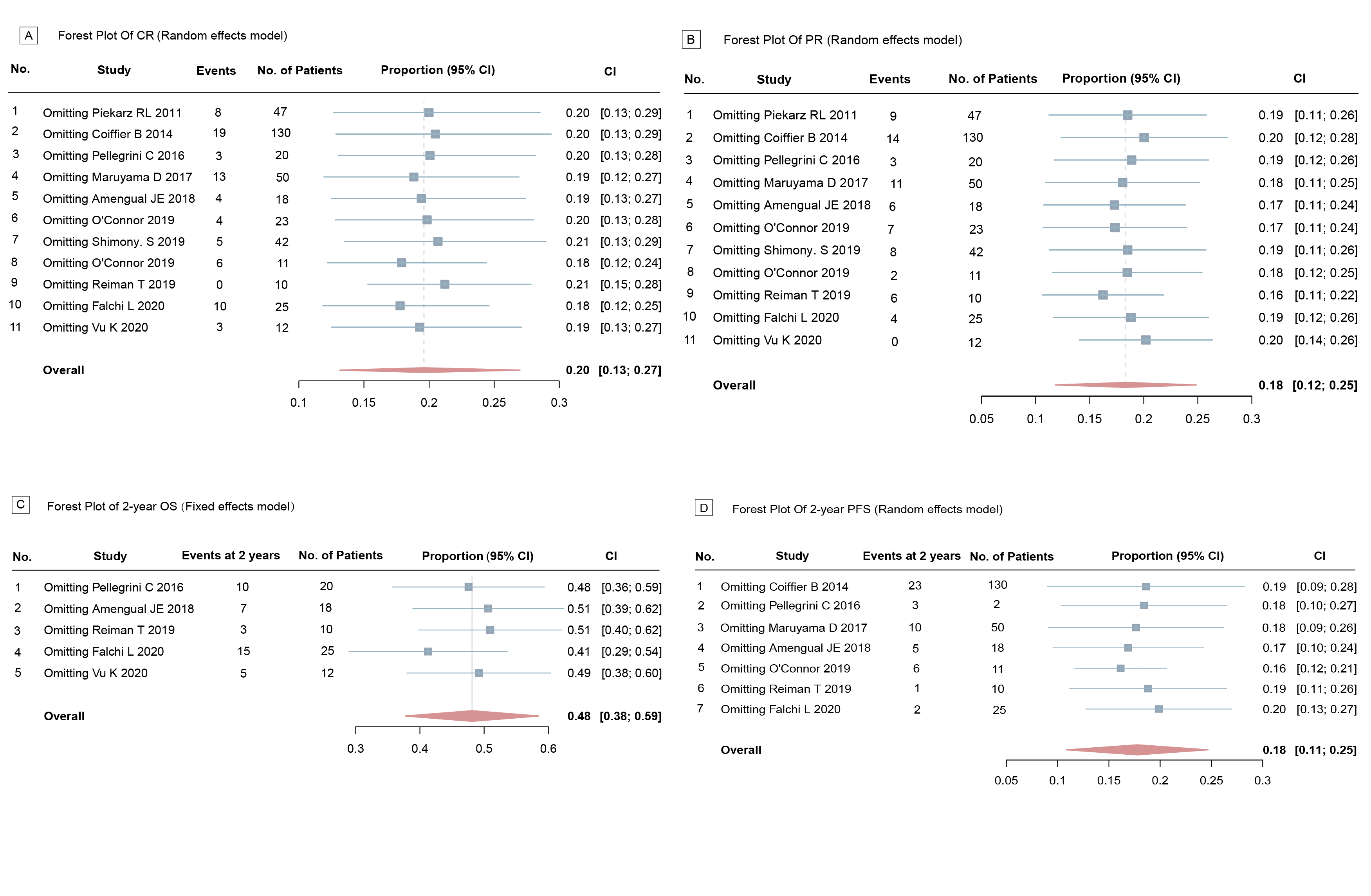

Supplement: Supplementary Figure S1 — Forest plots for sensitivity analysis of CR, PR, 2-year OS and 2-year PFS. (A) Forest plot of CR (random effects model); (B) Forest plot of PR (random effects model); (C) Forest plot of 2-year OS (fixed effects model); (D) Forest plot of 2-year PFS (random effects model). [file Image_1.TIF]

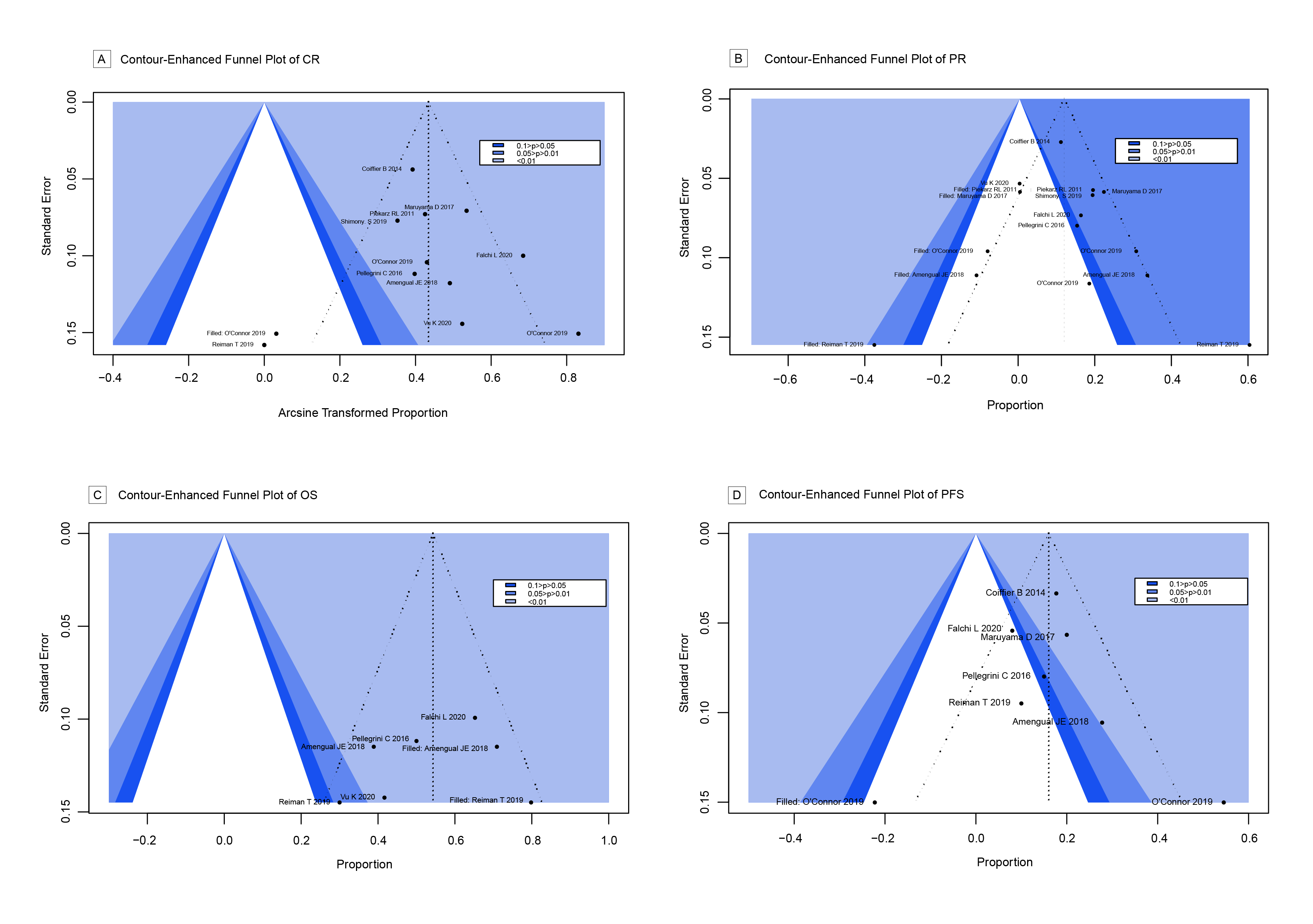

Supplement: Supplementary Figure S2 — Contour-enhanced funnel plots for sensitivity analysis of CR, PR, 2-year OS and 2-year PFS. (A) Contour-enhanced funnel plot for complete response (CR); (B) Contour-enhanced funnel plot for partial response (PR); (C) Contour-enhanced funnel plot for 2-year overall survival (OS); (D) Contour-enhanced funnel plot for 2-year progression-free survival (PFS). [file Image_2.TIF]

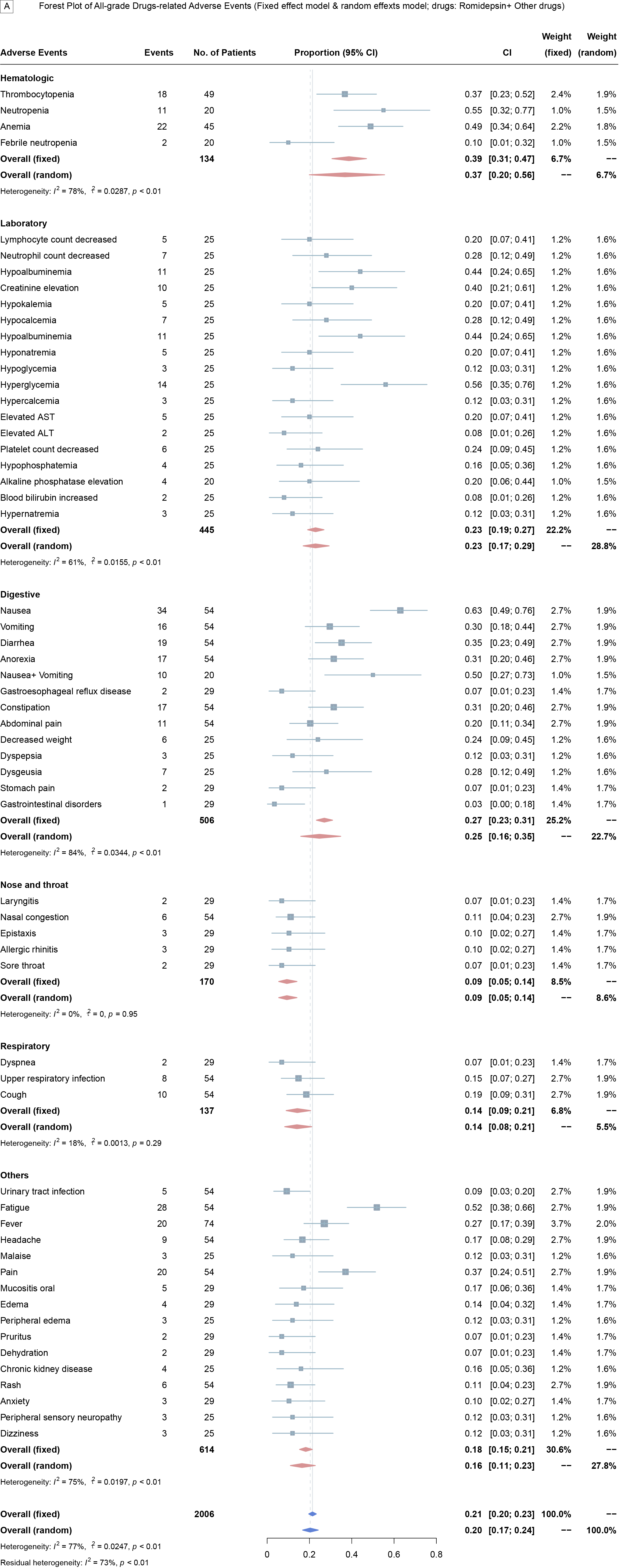

Supplement: Supplementary Figure S3 — Forest Plots for Over Proportion of Adverse Events in treatment with romidepsin combined with other drugs. (A) Proportion of all-grade adverse events in treatment with romidepsin combined with other drugs; (B) Proportion of grade 3 or higher adverse events in treatment with romidepsin combined with other drugs. [file Image_3.TIF]

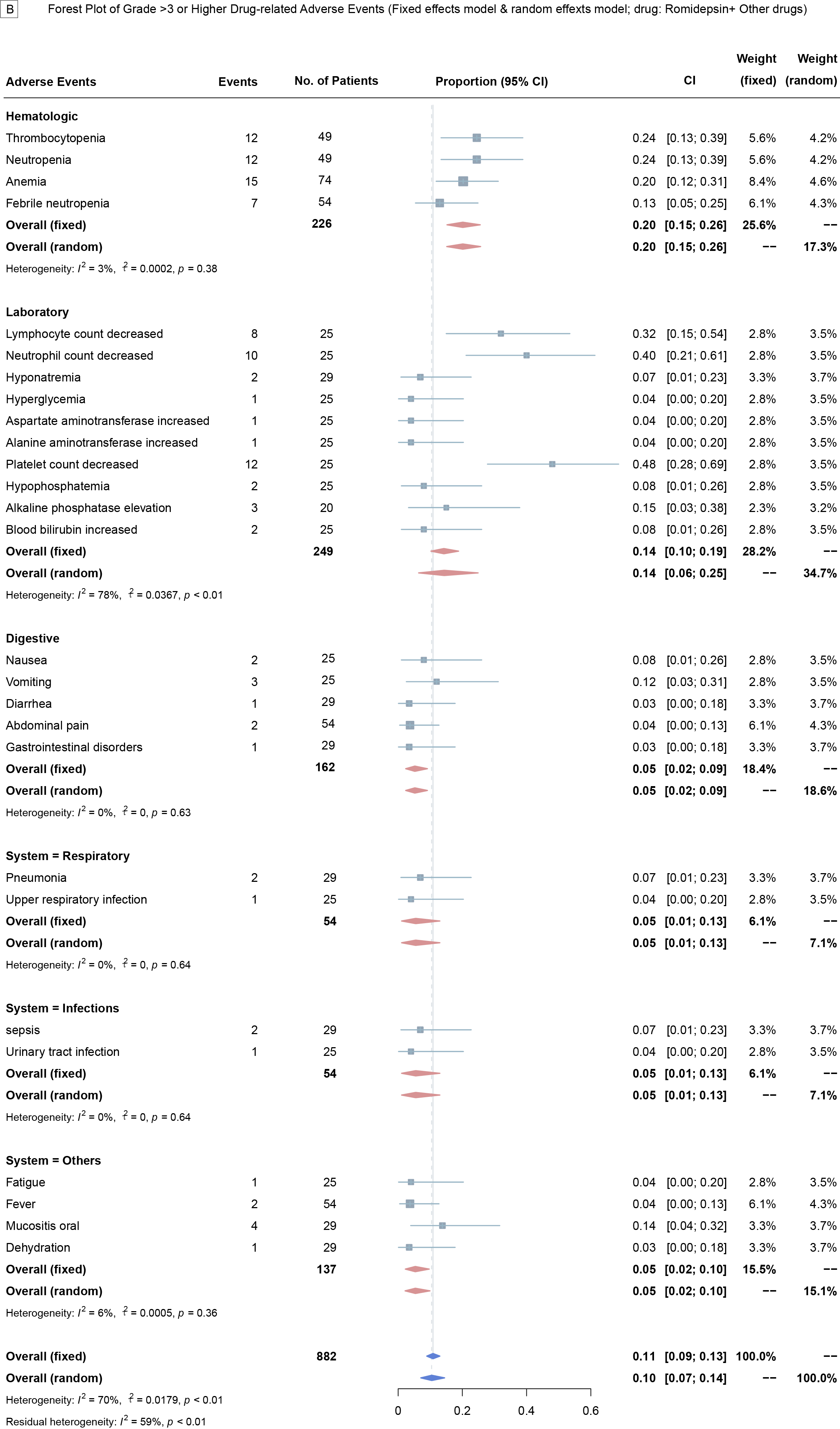

Supplement: Supplementary file 4 [file Image_4.TIF]
